# Temporal comparison of radiological and functional outcomes in calcaneal fracture surgery with and without iliac crest graft application: Mid- to long-term results

**DOI:** 10.1007/s00068-024-02687-5

**Published:** 2025-01-14

**Authors:** Enes Polat, Muhammed Yusuf Afacan, Bedri Karaismailoglu, Huseyin Botanlioglu, Ali Seker

**Affiliations:** Cerrahpasa Faculty of Medicine, Department of Orthopaedics and Traumatology, Istanbul University-Cerrahpasa, Istanbul, Turkey

**Keywords:** Calcaneus fractures, Open reduction with plate and screw fixation, Autograft usage, VAS score, AOFAS score, Subtalar arthrosis, Mid-to long-term follow-up

## Abstract

**Background:**

The standard approach for addressing intra-articular calcaneal fractures involves open reduction with plate and screw fixation, with ongoing discourse regarding the application of grafts to address bone gaps. The aim of this study is the temporal comparison of the radiological and functional outcomes in patients undergoing surgery for intra-articular calcaneal fractures, with a specific focus on the use of bone grafts.

**Methods:**

Thirty patients, comprising 13 with iliac grafts and 17 without, were enrolled in the study. Preoperative and postoperative assessments included Gissane and Böhler angles, Visual Analog Scale (VAS) scores, American Orthopedic Foot and Ankle Society (AOFAS) ankle hindfoot scale, and Kellgreen-Lawrance subtalar arthrosis stages. The average follow-up period was 6.7 years, ranging from a minimum of 3.5 to a maximum of 10 years.

**Results:**

The Böhler angle exhibited a significant increase (*p* < 0.001), while the Gissane angle did not show significant changes in the early postoperative period across the entire study group (*p* = 0.1). Graft-treated patients demonstrated a significantly higher Böhler angle in the early and late postoperative periods compared to preoperative values (*p* = 0.04, *p* = 0.05). Similarly, patients without grafts exhibited a significantly higher Böhler angle in the early and late postoperative periods compared to preoperative values (*p* = 0.004, *p* = 0.002). No significant differences were observed between periods in Gissane measurements (*p* = 0.3), VAS scores, AOFAS scores, and the development of subtalar arthrosis in both grafted and non-grafted patients.

**Conclusions:**

Evaluation of patients with calcaneal fractures, both with and without grafts, was conducted using Böhler and Gissane angles, VAS scores, AOFAS scores, and the development of fracture union and subtalar arthrosis, assessing preoperative, early, and late postoperative periods. No significant differences were found between the two groups in terms of clinical and radiological outcomes during mid-to long-term follow-up.

**Level of evidence:**

A retrospective cohort study.

**Supplementary Information:**

The online version contains supplementary material available at 10.1007/s00068-024-02687-5.

## Introduction

One of the prevailing injuries among tarsal bones is calcaneus fractures, a notable concern owing to their impact on the lives of young, active individuals, with potential socioeconomic and sociocultural ramifications. Intra-articular fractures, constituting 60–75% of calcaneal fractures, particularly those that are displaced, are associated with elevated morbidity [1, 2].

The established approach for addressing intra-articular calcaneal fractures involves open reduction with plate and screw fixation. However, the incorporation of bone grafts in calcaneus fracture surgeries remains a subject of contention, lacking robust evidence supporting discernible functional benefits [3]. Proponents argue that bone grafts may facilitate fracture healing, enable early weight-bearing, mitigate post-traumatic arthritis, and avert delayed collapse by enhancing mechanical strength [4, 5]. Conversely, it is posited that the highly vascular nature of the calcaneus allows for radiological healing within 4–8 weeks without the use of bone grafts, with internal fixation adequately supporting the articular surface. Moreover, concerns regarding heightened infection rates, blood loss, postoperative pain, and specific issues related to donor site morbidity and complications underscore the nuanced decision-making surrounding bone grafting [4, 6–8].

This study seeks to juxtapose the radiological and functional outcomes of patients who underwent surgery for intra-articular calcaneal fractures with and without the use of bone grafts. The main objective is to analyze radiological and functional outcomes over time within each treatment group, with some comparative assessment between groups and to discern whether the inclusion of grafts in surgery confers advantages over patients devoid of grafts during mid-to long-term follow-up.

## Materials and methods

**Fig. 1:**
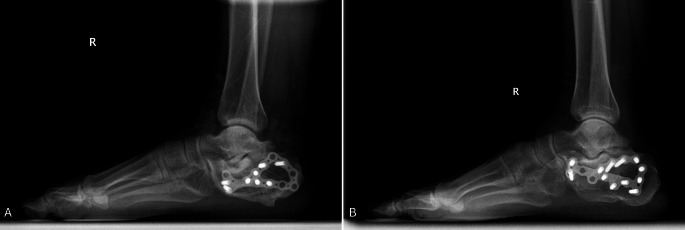
Anatomical calcaneus plates featuring low-profile designs and locking screws were used for permanent fixation in both grafted (**1A**) and not-grafted (**1B**) patients

Thirty adult patients who underwent surgery for displaced intra-articular calcaneal fractures were enrolled in the study. Thirteen patients (43.3%) received an iliac wing graft during the operation, while 17 patients (56.7%) did not. Retrospective evaluation of patients’ files was conducted as part of the study. A total of 320 patient files were examined, and individuals with open calcaneal fractures, pathological calcaneal fractures, those without a surgical indication, those under 18 years of age, those with a duration of less than 3 years of follow-up, as well as those who do not wish to participate in the work were excluded. Thus, only 30 patients met the study’s inclusion criteria and were selected for final analysis. The average follow-up period was 6.7 (range, 3.5–10) years. In this study “early follow-up” refers to assessments conducted within the first few months post-surgery, “mid-term follow-up” denotes evaluations between 3 and 5 years, and “long-term follow-up” encompasses evaluations beyond 5 years. The surgical interventions were carried out by two distinct orthopedic surgeons, with one utilizing iliac wing grafts in all calcaneal fractures and the other refraining from their application. A surgical technique employing an extended lateral approach was employed for both groups. Following the reduction of the posterior articular surface, the defect was filled with autograft from the iliac wing in one group, while the other group did not receive graft support to maintain calcaneal height. Both groups underwent fixation with anatomical calcaneus plates featuring low-profile designs and locking screws after reduction (see Fig. 1).

As a postoperative follow-up protocol, patients in both groups were mobilized without bearing weight for the initial 8 weeks after surgery. Subsequently, for the following 2 weeks, partial weight-bearing was introduced. From the completion of the 10th week after surgery onward, patients were mobilized with full weight-bearing. Thus, the impact of postoperative mobilization and weight-bearing on the outcomes was minimized for patients. During clinical assessments, patients underwent follow-up physical examinations. In addition to acquiring anteroposterior and lateral images of the foot and ankle, foot oblique and axial (Harris) radiographs were taken. The Böhler and Gissane angles were subsequently measured from the lateral radiograph. In the course of patient evaluation, the American Orthopedic Foot and Ankle Society (AOFAS) hindfoot scores were computed during the control examinations. The nine-item scoring system encompassed pain, limitations, gait style, walking distance, walking surface, and results from the physical examination, including the range of motion, stability, and alignment of the ankle and hindfoot joint. In the calculation of the AOFAS hindfoot score, objective measurements involved the use of a goniometer to assess the range of motion (ROM) of the ankle and subtalar joint. In cases of unilateral fractures, the contralateral foot served as a control, whereas normal values were employed as a reference in bilateral fractures. Visual Analogue Scale (VAS) pain scores for patients were meticulously recorded and graded on a scale ranging from 0 to 10 points, with 10 points representing the utmost severity of pain. The Kellgren-Lawrance subtalar arthrosis staging, ranging from 0 to 4, was conducted. Both preoperative and postoperative assessments included the examination of Gissane and Böhler angles, VAS scores, AOFAS ankle hindfoot scale, and Kellgren-Lawrance subtalar arthrosis stages, allowing for a comprehensive comparative analysis. All procedures performed in studies involving human participants were in accordance with the ethical standards of the institutional and/or national research committee and with the 1964 Helsinki declaration and its later amendments or comparable ethical standards. Ethical committee approved the study.

Descriptive data were expressed as mean, standard deviation, median for continuous variables, and frequency and percentage for categorical variables. Normal distribution of continuous variables was assessed using histogram, Kolmogrov-Smirnov, and Shapiro-Wilks tests, while Levene’s t-test was employed for evaluating group homogeneity. Non-parametric values were analyzed using the Mann-Whitney U test, and the chi-square test assessed categorical distribution. Friedman and Wilcoxon Signed Ranks tests were applied to analyze the distribution of preoperative and postoperative Böhler and Gissane Angles within the research group. All statistical analyses employed a Type 1 error (*p* < 0.05) as the threshold for significance and were conducted using the SPSS 26.0 (IBM) package program.

## Results

The average age of the participants in our study cohort was 44.4 ± 13.6 years, with comparable ages observed in both the grafted and untreated groups (*p* = 0.9). Among the participants, 21 (70%) were male, while 9 (30%) were female, and the gender distribution exhibited similarity between the groups (*p* = 0.7) (refer to Table 1).

**Table 1:** Descriptive characteristics of the Research Group (n:30)

	Whole group		Graft +		Graft -		
	**Average ± SD**	**Median**	**Average ± SD**	**Median**	**Average ± SD**	**Median**	**p**
Age (year)	44.43 ± 13.62	47.0	48.23 ± 11.73	45.0	50.35 ± 15.19	49.0	0.902^a^
Gender	**n**	**%**	**n**	**%**	**n**	**%**	
Male	21	70.0	10	76.9	11	64.7	0.691^b^
Female	9	30.0	3	23.1	6	35.3	
Total	30	43.3	13	56.7	17	100	

^a^Mann-Whitney U Test, ^b^Chi-square test

n: Number

SD: Standart Deviation

Graft +: Iliac Wing Graft Applied

Graft -: Iliac Wing Graft not Applied

p: significance level (0.05)

Notably, significant differences were observed in the preoperative, early postoperative, and late postoperative changes in Böhler and Gissane measurements between the groups (*p* < 0.001) (see Table 2). Analyzing the preoperative and early postoperative Böhler and Gissane angles within the study group revealed a significant increase in the Böhler angle during the early postoperative period (*p* < 0.001), while the Gissane angle did not exhibit a significant change (*p* = 0.10) (Table 2). Furthermore, the early postoperative and late postoperative Böhler and Gissane angles in the study group displayed similarity (*p* = 0.98, *p* = 0.22) (Table 2). Comparison of preoperative, early postoperative, and late postoperative Böhler and Gissane angles in patients with and without iliac wing graft revealed no significant differences (Table 2).

**Table 2:** Evaluation and distribution of preoperative, early, and late postoperative BÖHLER, GISSANE measurements in the entire patient group (n:30)

	Böhler	Gissane
*Preoperative*		
Average ± SD	13.73 ± 13.50	111.30 ± 21.60
Median	13.50	114.50
Minimum-Maximum	-27.0–40.0	52–150
*Early Postoperative*		
Average ± SD	24.76 ± 8.63	117.40 ± 9.56
Median	24.50	115.0
Minimum-Maximum	0.0–40.0	95–135
p^a^ pre-op, early post-op	**< 0.001**	0.975
*Late Postoperative*		
Average ± SD	27.70 ± 18.69	113.60 ± 20.09
Median	25.0	115.0
Minimum-Maximum	0–115	20–132
p^a^ early, late post-op	0.101	0.219
p^b^	**< 0.001**	0.159
*Healthy*		
Average ± SD	29.30 ± 5.26	
Median	30	
Minimum-Maximum	20–45	
p^a^ early post-op, healthy	**0.008**	
p^a^ late post-op, healthy	**0.020**	

^a^Wilcoxon Test

^**b**^Friedman Test of pre-op, early and late post-op

SD: Standart Deviation

p: significance level (0.05)

However, in graft-treated patients, the distribution of preoperative, early postoperative, and late postoperative measurements indicated a significant change in the Böhler angle (*p* = 0.04), while no difference was observed in the Gissane measurements between early and late postoperative periods (*p* = 0.3) (Table 3). The Böhler angle in graft-treated patients was significantly higher in both the early postoperative and late postoperative periods compared to the preoperative mean values (*p* = 0.04, *p* = 0.05) (Table 3). Similarly, in patients without grafts, significant changes were noted in the Böhler angle (*p* = 0.002), with no significant difference in Gissane measurements between early and late postoperative periods (*p* = 0.3) (Table 3). The Böhler angle in patients without grafts was significantly higher in both the early postoperative and late postoperative periods compared to the preoperative mean values (*p* = 0.004, *p* = 0.002) (Table 3).

**Table 3:** Distribution of preoperative, early and late postoperative measurements in patient groups with and without iliac wing grafts

Graft + (*n*:13)				Graft - (*n*:17)			
**BÖHLER ANGLE MEASUREMENTS**							
	**Average ± SD**	**Median**	**Min-Max**	**Average ± SD**	**Median**	**Min-Max**	**p** ^**c**^
Pre-op BÖHLER	15.76 ± 12.17	15.0	-4-40	12.17 ± 15.09	12.0	-27-36	0.680
Early Post-op BÖHLER	25.84 ± 7.18	25.0	16–38	23.94 ± 9.73	24.0	0–40	0.457
**p** ^**a**^ ** pre-op and early post-op 0.04**				**p** ^**a**^ ** pre-op and early post-op 0.004**			
Late Post-op BÖHLER	32.53 ± 26.25	26.0	8-115	24.00 ± 9.10	25.0	0–40	0.680
**p** ^**a**^ ** early and late post-op 0.916**				**p**^**a**^** early and late post-op** 0.997			
**p** ^**a**^ ** pre-op and late post-op 0.045**				**p** ^**a**^ ** pre-op and late post-op 0.002**			
p^b^	**0.038**				**0.002**		
Healthy BÖHLER	29.30 ± 7.68	27.0	20–45	29.29 ± 2.44	30.0	25–35	0.990
**p** ^**a**^ ** early post-op and healthy 0.264**							
**p** ^**a**^ ** late post-op and healthy 0.614**							
**GISSANE ANGLE MEASUREMENTS**							
Pre-op GISSANE	104.92 ± 28.09	108.0	52–150	116.17 ± 14.00	115.0	94–140	0.408
Early Post-op GISSANE	117.07 ± 13.10	115.0	95–135	117.64 ± 6.07	115.0	105–128	0.967
Late Post-op GISSANE	109.84 ± 29.38	115.0	20–132	116.47 ± 8.00	115.0	100–130	0.408
**p** ^**b**^	0.303				0.276		
Healthy GISSANE	118.07 ± 8.54	120.0	100–135	120.17 ± 9.80	125.0	103–130	0.363

^a^Wilcoxon Test

^b^Friedman Test of pre-op, post-op, and control

^c^Mann-Whitney U Test

SD: Standart Deviation

pre-op: Before the operation

post-op: After the operation

Min: Minimum value, Max: Maksimum value

p: Significance level (0.05)

Across the entire cohort, measurements on the healthy side were significantly higher than early postoperative and late postoperative Böhler measurements (*p* = 0.008, *p* = 0.02) (Table 2). Moreover, there were no significant differences between the grafted and non-grafted groups in postoperative follow-up years, VAS, AOFAS scores, development of subtalar arthrosis, and Sanders types (Table 4).

**Table 4:** Distribution of Post-op follow-up period in Year, AOFAS, VAS, Subtalar Arthrosis and Sanders Variables

	Whole group		Graft +		Graft -		
	**Average ± SD**	**Median**	**Average ± SD**	**Median**	**Average ± SD**	**Median**	**p**
Post-op year	6.70 ± 2.98	7.5	7.00 ± 4.16	9.0	6.47 ± 1.73	6.0	0.363^a^
AOFAS	76.73 ± 14.93	80.0	80.38 ± 14.10	83.0	73.94 ± 15.35	77.0	0.133^a^
VAS	3.96 ± 1.71	4.0	3.61 ± 1.98	3.0	4.23 ± 1.48	4.0	0.213^a^
Subtalar Arthrosis	**n**	**%**	**n**	**%**	**n**	**%**	
Type2	11	36.7	6	46.2	5	29.4	0.394^b^
Type3	11	36.7	3	23.1	8	47.1	
Type4	8	26.6	4	30.7	4	23.5	
Sanders							
Type3	13	43.3	8	61.5	5	29.4	0.138^b^
Type4	17	56.7	5	38.5	12	70.6	
Total	30	100	13	100	17	100	

SD: Standart Deviation

^a^Mann-Whitney U

^b^Chi-square

p: Significance level (0.05)

## Discussion

This study demonstrated that there were no discernible differences in both radiological and clinical outcomes concerning graft utilization. Furthermore, the preservation of calcaneus morphology was observed in the postoperative period, irrespective of the presence or absence of grafts.

The Böhler angle decreases after calcaneal fractures which subsequently increases after reduction. Monitoring the Böhler angle after surgery is crucial for assessing reduction stability. Sugimoto et al. underscored the significance of maintaining the Böhler angle at standard values postoperatively, which was initially low. They also emphasized that the significance in terms of reduction depended on not exhibiting a subsequent decrease in the follow-ups [9]. Longino et al. demonstrated an improvement in the Böhler angle post-surgery, with minimal decline during follow-ups [10]. Barroco et al. explored the postoperative reproducibility of the Böhler and Gissane angles, highlighting the greater ease of restoring the Böhler angle compared to the Gissane angle [11]. Our study revealed a significant enhancement in the postoperative Böhler angle within the entire patient group compared to the preoperative period. However, no substantial difference was observed in the Gissane angle. In both treatment methods, there was no significant difference between the early and late postoperative periods for both angle measurements. Consequently, the Böhler angle reached standard values postoperatively in both treatment groups, indicating the preservation of reduction. Notably, there was no decline in the Böhler angle during the early and late postoperative periods in both groups, affirming the sustained reduction. Conversely, the Gissane angle exhibited greater difficulty in restoration [11], consistent with previous findings.

Longino et al. conducted a study comparing postoperative Böhler angle degrees in 40 patients with calcaneal fractures, with and without grafts, finding no significant difference in angle elevation between the two groups [10]. Cao et al. investigated the use of grafts in calcaneal fractures in a study of 50 patients and reported no significant difference in Böhler angle during follow-up between the graft and non-graft groups [12]. Meta-analyses by Tian et al. [13] and Zheng et al. [14] also failed to identify significant differences in Böhler and Gissane angles or postoperative complications between patients with and without grafts although the clinical scores were better in patients with graft in the meta-analysis by Zheng et al. [14].

Despite these findings, some publications suggest a radiological difference with the use of grafts. Yang et al.‘s systematic review concluded that while bone grafts did not significantly impact function, they improved Böhler angle restoration, enabling earlier full weight-bearing in patients with grafts [15]. In studies by Duymus et al. [16] and Singh et al. [2], the use of bone allografts led to better restoration of the Böhler angle, allowing for earlier weight-bearing. However, the clinical results and complications were similar [2].

Our study, analyzing patient groups with and without iliac wing grafts separately, demonstrated a significant increase in the Böhler angle in both groups during early and late postoperative periods compared to the preoperative period. However, no significant difference was observed between the two groups, and the Gissane angle remained unchanged in both groups. While restoring radiological parameters is crucial in calcaneus fractures, assessing the proximity of these angles to healthy side measurements post-fracture healing is equally important, particularly in the last follow-up.

As per some authors, the preoperative assessment of the Böhler angle serves as an indicator for the severity of a fracture, while the postoperative measurement of the Böhler angle is indicative of functional recovery outcomes. In an epidemiological study encompassing 752 fractured sides, Mitchell et al. established a significant correlation between fracture severity and the preoperative reduction in the Böhler angle [1]. A study conducted by Su et al., involving 274 patients, compared the Böhler angle of the unaffected calcaneus with that of the fractured side post-surgery. Although a postoperative disparity persisted between measurements on the intact side and the fractured side, the results of postoperative assessments approached those of the unaffected side. Furthermore, it was observed that the preoperative Böhler angle is more closely associated with fracture severity, whereas the postoperative Böhler angle is more closely associated with functional outcomes [17]. That was an indication of improved functional results. In another study of 44 patients by Paley et al., the fractured side measurements were lower compared to the intact side [18].

In our study, Böhler measurements on the healthy side within the entire group were significantly higher than the early and late postoperative Böhler measurements. Despite the similarity between the Böhler angle measurements of intra-articular calcaneal fractures, both with and without grafts, and those of the healthy side in early and late postoperative assessments, such uniformity was not observed between the two methods when compared to the healthy side. Consequently, the graft’s superiority in angle restoration could not be substantiated. It is noteworthy that while the Böhler angle of the fractured side approached the measurements of the healthy side after surgery and during controls, indicating success in both treatment methods, neither demonstrated superiority over the other.

In the analysis of postoperative outcomes in the literature for groups with and without grafts in open reduction and internal fixation of calcaneus fractures, various parameters such as VAS, AOFAS scores, and the development of subtalar arthrosis in patient follow-ups were utilized. Sanders et al. conducted a 10-20-year follow-up on 108 patients without grafts who underwent open reduction and internal fixation with an unlocked plate. They assessed AOFAS and VAS scores, as well as the need for subtalar arthrodesis, questioning the necessity of grafts and the adequacy of the unlocked lateral plate. The results indicated that neither grafts nor locking plates were necessary as there was no loss of reduction during the patient follow-up. Moreover, patients experiencing subtalar arthrosis exhibited significant pain symptoms, discernible through monitoring the VAS score [19]. In a study by Longino et al., a comparison of grafted and non-grafted groups in postoperative VAS scores revealed no significant difference [10].

Su et al. showed that the postoperative improvement in Böhler’s angle positively affected the postoperative AOFAS score, which increased clinical improvement [17]. Schepers et al. questioned the AOFAS score, the development of secondary arthrosis, and the need for arthrodesis in the follow-up of clinical results. As a result, open reduction and internal fixation methods prevailed over conservative treatment and percutaneous procedures, and secondary arthrodesis was performed in 20% of patients, while the nationwide use of the AOFAS score was 7% [20]. According to the systematic review of Yang et al., the AOFAS score of the group using bone graft was lower than the group without bone graft [15].

According to the comparison results of the grafted and non-grafted groups in the study of Duymus et al., including 43 calcaneal fractures, although the radiological results were more satisfactory in the graft-used group, the AOFAS score, and the clinical results were similar in both groups [16].

In a study of 390 cases by Singh et al., AOFAS scores were similar in both groups, while the radiological results of the grafted group were better [2]. In a study of 57 cases by Cao et al., no significant difference was found in the follow-ups of the grafted and non-grafted groups in terms of AOFAS scores and Böhler, Gissane angles [12]. In the meta-analysis of Tian et al., there was no significant difference between the two groups in terms of AOFAS scores, Böhler and Gissane angle measurements, and postoperative wound infection development between the grafted and non-grafted groups [13]. However, in the meta-analysis of Zheng et al., although the Böhler and Gissane angles were the same in the grafted and untreated groups, the postoperative AOFAS score was better in the bone-grafted group [14].

Studies show the effect of age and gender on the prognosis of calcaneal fractures in the literature. Tufescu et al. emphasized that the male gender harms the healing of calcaneal fractures, returning to work is later in men compared to women, and the work capacity in men is not the same as before [21]. However, a multicenter, retrospective, and more recent study by Sugimoto et al. showed that the female gender is a risk factor for increased reduction loss in fractures [9]. A study investigating the effect of age on prognosis in calcaneal fractures showed that clinical outcomes were better in elderly patients, while foot functions were better in younger patients [22]. In our study, almost half of the participants were graft-treated. The fact that the mean age and gender of patients with and without grafts were similar in our research eliminated the effect of age and gender on the results.

The decision to use graft was based on the operating surgeon’s preference. One surgeon consistently utilized iliac crest grafts while the other did not. This introduces potential allocation bias, which could impact study outcomes. Acknowledged that the study’s applicability to other surgical teams or graft materials may be limited due to the specific practices of the two surgeons. On the other hand, this does not prevent us from comparing the early and long-term results within each group independently. In our study, we evaluated 30 patients. There are studies with a similar and larger number of patients in the literature with similar results. Although this study may show more accurate results with a larger number of patients, the fact that the groups with and without grafts were close to each other in terms of numbers, and that there was no significant difference between them in terms of age, gender and follow-up period shows the reliability of the study. However, although our average follow-up period is 6.7 years, subtalar arthrosis stages may be lower due to the shorter follow-up period of some patients. However, this does not constitute a significant limitation as it has the same effect in both groups. In addition, the decision to use grafts during the operation was left to the surgeon’s preference, and no specific preference was used in this decision. Moreover, this study is valuable because of demonstrating mid-to long-term follow-up results.

## Conclusion

As a result, in our study, Böhler, Gissane angles, VAS, AOFAS scores, and subtalar arthrosis development were determined in the preoperative, early and late postoperative periods of patients with intra-articular displaced calcaneal fractures, with and without grafts. There was no significant difference between the two groups in terms of clinical and radiological results at mid-to long-term follow-up. In the light of these results, during open reduction and internal fixation of calcaneal fractures with intra-articular extension, procedures such as invasive graft removal to the iliac wing and intraoperative graft placement in the calcaneal fracture will prolong the operation time and increase the infection formation with the extra graft removal procedure and cause additional donor site complications. Therefore, we do not recommend bone grafting. The graft application procedure does not have a significant contribution to the clinical and radiological results of the patient at mid-to long-term follow-up. Additional studies with larger, multicenter samples and randomized protocols are essential for confirming these findings across diverse clinical settings and surgical practices.

## Electronic supplementary material

Below is the link to the electronic supplementary material.

Supplementary Material 1

## Data Availability

The data that support the findings of this study are available from the corresponding author, M.Y.A. upon reasonable request.
