# Supplementary material for: Temporal comparison of radiological and functional outcomes in calcaneal fracture surgery with and without iliac crest graft application: Mid- to long-term results
Source: Eur J Trauma Emerg Surg. 2025 Jan 14;51(1):15. doi: 10.1007/s00068-024-02687-5 (PMC11732894; doi:10.1007/s00068-024-02687-5)
Supplement: Supplementary file 1 — Supplementary Material 1 [file 68_2024_2687_MOESM1_ESM.docx]

**Table 1.** Descriptive Characteristics of the Research Group (n:30)

|  | Whole group | | Graft + | | Graft - | |  |
| --- | --- | --- | --- | --- | --- | --- | --- |
|  | **Average ± SD** | **Median** | **Average ± SD** | **Median** | **Average ± SD** | **Median** | **p** |
| Age (year) | 44.43±13.62 | 47.0 | 48.23±11.73 | 45.0 | 50.35±15.19 | 49.0 | 0.902^a^ |
| Gender | **n** | **%** | **n** | **%** | **n** | **%** |  |
| Male | 21 | 70.0 | 10 | 76.9 | 11 | 64.7 | 0.691^b^ |
| Female | 9 | 30.0 | 3 | 23.1 | 6 | 35.3 |  |
| Total | 30 | 43.3 | 13 | 56.7 | 17 | 100 |  |

^a^Mann-Whitney U Test, ^b^Chi-square test

n: Number

SD: Standart Deviation

Graft +: Iliac Wing Graft Applied

Graft -: Iliac Wing Graft not Applied

p: significance level (0.05)

**Table 2.** Evaluation and distribution of preoperative, early,

and late postoperative BÖHLER, GISSANE

measurements in the entire patient group (n:30)

| *Preoperative* | BÖHLER | GISSANE |
| --- | --- | --- |
| Average ± SD | 13^.^73±13.50 | 111.30±21.60 |
| Median | 13.50 | 114.50 |
| Minimum-Maximum | -27.0**-**40.0 | 52-150 |
| *Early Postoperative* |  |  |
| Average ± SD | 24.76 ±8.63 | 117.40±9.56 |
| Median | 24.50 | 115.0 |
| Minimum-Maximum | 0.0-40.0 | 95-135 |
| p^a^ pre-op, early post-op | **<0.001** | 0.975 |
| *Late Postoperative* |  |  |
| Average ± SD | 27.70±18.69 | 113.60±20.09 |
| Median | 25.0 | 115.0 |
| Minimum-Maximum | 0-115 | 20-132 |
| p^a^ early, late post-op | 0.101 | 0.219 |
| p^b^ | **<0.001** | 0.159 |
| *Healthy* |  |  |
| Average ± SD | 29.30±5.26 |  |
| Median | 30 |  |
| Minimum-Maximum | 20-45 |  |
| p^a^ early post-op, healthy | **0.008** |  |
| p^a^ late post-op, healthy | **0.020** |  |

^a^Wilcoxon Test

**^b^**Friedman Test of pre-op, early and late post-op

SD: Standart Deviation

p: significance level (0.05)

**Table 3.** Distribution of preoperative, early and late postoperative measurements in patient groups with and without iliac wing grafts

|  | Graft + (n:13) | | | Graft - (n:17) | |  |  |
| --- | --- | --- | --- | --- | --- | --- | --- |
| BÖHLER ANGLE MEASUREMENTS | | | | | | | |
|  | **Average ± SD** | **Median** | **Min-Max** | **Average ± SD** | **Median** | **Min-Max** | **p^c^** |
| Pre-op BÖHLER | 15.76±12.17 | 15.0 | -4-40 | 12.17±15.09 | 12.0 | -27-36 | 0.680 |
| Early Post-op BÖHLER | 25.84±7.18 | 25.0 | 16-38 | 23.94±9.73 | 24.0 | 0-40 | 0.457 |
| p^a^ pre-op and early post-op 0.04 | | | | **p^a^ pre-op and early post-op** **0.004** | | | |
| Late Post-op BÖHLER | 32.53±26.25 | 26.0 | 8-115 | 24.00±9.10 | 25.0 | 0-40 | 0.680 |
| p^a^ early and late post-op 0.916 | | | | **p^a^ early and late post-op** 0.997 | | | |
| p^a^ pre-op and late post-op 0.045 | | | | **p^a^ pre-op and late post-op** **0.002** | | | |
| p^b^ | **0.038** |  |  |  | **0.002** |  |  |
| Healthy BÖHLER | 29.30±7.68 | 27.0 | 20-45 | 29.29±2.44 | 30.0 | 25-35 | 0.990 |
| p^a^ early post-op and healthy 0.264 | | | |  |  |  |  |
| p^a^ late post-op and healthy 0.614 | | | |  |  |  |  |
| GISSANE ANGLE MEASUREMENTS | | | | | | | |
| Pre-op GISSANE | 104.92±28.09 | 108.0 | 52-150 | 116.17±14.00 | 115.0 | 94-140 | 0.408 |
| Early Post-op GISSANE | 117.07±13.10 | 115.0 | 95-135 | 117.64±6.07 | 115.0 | 105-128 | 0.967 |
| Late Post-op GISSANE | 109.84±29.38 | 115.0 | 20-132 | 116.47±8.00 | 115.0 | 100-130 | 0.408 |
| p^b^ | 0.303 |  |  |  | 0.276 |  |  |
| Healthy GISSANE | 118.07±8.54 | 120.0 | 100-135 | 120.17±9.80 | 125.0 | 103-130 | 0.363 |

^a^Wilcoxon Test

^b^Friedman Test of pre-op, post-op, and control

^c^Mann-Whitney U Test

SD: Standart Deviation

pre-op: Before the operation

post-op: After the operation

Min:Minimum value, Max: Maksimum value

p: Significance level (0.05)

**Table 4.** Distribution of Post-op Follow-up Period in Year, AOFAS, VAS, Subtalar Arthrosis and Sanders Variables

|  | Whole Group | | | Graft + | | Graft - | |  |
| --- | --- | --- | --- | --- | --- | --- | --- | --- |
|  | **Average ± SD** | | **Median** | **Average ± SD** | **Median** | **Average ± SD** | **Median** | **p** |
| Post-op year | | 6.70±2.98 | 7.5 | 7.00±4.16 | 9.0 | 6.47±1.73 | 6.0 | 0.363^a^ |
| AOFAS | 76.73±14.93 | | 80.0 | 80.38±14.10 | 83.0 | 73.94±15.35 | 77.0 | 0.133^a^ |
| VAS | 3.96±1.71 | | 4.0 | 3.61±1.98 | 3.0 | 4.23±1.48 | 4.0 | 0.213^a^ |
| Subtalar Arthrosis | **n** | | **%** | **n** | **%** | **n** | **%** |  |
| Type2 | 11 | | 36.7 | 6 | 46.2 | 5 | 29.4 | 0.394^b^ |
| Type3 | 11 | | 36.7 | 3 | 23.1 | 8 | 47.1 |  |
| Type4 | 8 | | 26.6 | 4 | 30.7 | 4 | 23.5 |  |
| Sanders |  | |  |  |  |  |  |  |
| Type3 | 13 | | 43.3 | 8 | 61.5 | 5 | 29.4 | 0.138^b^ |
| Type4 | 17 | | 56.7 | 5 | 38.5 | 12 | 70.6 |  |
| Total | 30 | | 100 | 13 | 100 | 17 | 100 |  |
|  |  |  |  |  |  |  |  |  |

SD: Standart Deviation

^a^Mann-Whitney U

^b^Chi-square

p: Significance level (0.05)
